# Traditional and complementary medicine use among chronic haemodialysis patients: a nationwide cross-sectional study

**DOI:** 10.1186/s12906-021-03268-4

**Published:** 2021-03-16

**Authors:** Nor Fadhlina Zakaria, Mohd Tawfeq Mohd Noor, Rafidah Abdullah

**Affiliations:** 1 Faculty of Medicine and Health Sciences, Universiti Putra Malaysia, 43400 Serdang, Selangor Malaysia; 2 Putrajaya Hospital, Jalan P9, Presint 7, 62250 Putrajaya, Malaysia

**Keywords:** Traditional complementary medicine, Haemodialysis, Prevalence

## Abstract

**Background:**

In the era of digital and improved conventional medicine, many continue to use traditional and complementary medicine (TCM). The prevalence of its usage is not well reported, especially in patients with end-stage kidney disease (ESKD) receiving haemodialysis, thus its benefits and adverse effects are not widely known. This study determines the prevalence, types, perceptions and factors associated with TCM use by chronic haemodialysis patients in Malaysia.

**Methods:**

This is a multi-centre cross-sectional study involving patients undergoing haemodialysis treatment in Malaysia. A validated face-to-face questionnaire-based interview was conducted. Sociodemographic and clinical profiles of the patients, factors associated with TCM use, perceptions, sources of information, and disclosures to treating doctors were obtained. Data were analysed using SPSS software.

**Results:**

A total of *n* = 329 participants were recruited. The mean age of the participants was 54.9 ± 12.5 years. The majority were Malays (72%) and females (54.7%). A total of 64.7% (*n* = 213) reported TCM use; *n* = 132 used TCM before the initiation of dialysis, while *n* = 81 used TCM after initiation. In the post-hoc analysis, patients who had never used TCM had a higher mean age (56.7 ± 12.3 years) than the patients who used TCM (51.1 ± 13.1) (*p* = 0.015) and were likely to have received primary education (*p* = 0.011). Unemployment was more likely to be associated with non-TCM use; with odds ratio 1.85 (95% CI: 1.15, 2.98). Biologically based therapy was found to be the most popular (97.2%) type of TCM, including herbal medicine (67.6%) and supplements (58.0%). Most respondents did not disclose their TCM use to their doctors (72.3%), and 41.8% had the perception that they felt better.

**Conclusions:**

TCM is widely used among chronic haemodialysis patients in Malaysia, mainly herbal medicine and supplements. Non-disclosure to healthcare professionals and a poor monitoring and regulation of its use in ESKD patients could be detrimental. Awareness needs to be raised among healthcare professionals and the general population.

**Trial registration:**

The Ethics Committee for Research, University Putra Malaysia (13th March 2019). Reference: UPM/TNCPI/RMC/1.4.18.2 (JKEUPM).

**Supplementary Information:**

The online version contains supplementary material available at 10.1186/s12906-021-03268-4.

## Background

Traditional medicine is used in the maintenance of health and in the prevention, diagnosis, improvement or treatment of physical and mental illness [1]. It is often termed alternative or complementary medicine; these terms have been used interchangeably. It refers to knowledge, skills and practises based on the theories, beliefs and experiences indigenous to different cultures [1]. The practice of traditional and complementary medicine (TCM), particularly in low- and middle-income countries, has been increasing over the past few years [2]. It is estimated that one-third of the population lacks access to essential medicines, thus explaining the rising prevalence of TCM [1]. In certain parts of the world, TCM could be the main or the only source of health care [3]. The growing trend for patients to take a more proactive approach to their own health and to seek out different forms of self-care is another reason for its usage [3]. TCM has been used in major parts of the world for the maintenance of health and well-being and is culturally accepted and trusted [1].

The reported global prevalence of TCM use ranges widely from 9.8 to 76.0% [4], while the prevalence of TCM use among Malaysians in their lifetime was 69.4% (67.6–71.2%) [5]. The use of TCM in Malaysia appears to have grown steadily over recent years, with herbal treatments reportedly being the most popular type. Worldwide, the use of herbal treatments was also reported to be the most prevalent form of traditional medicine [1]. Modalities of TCM in Malaysia are categorized into 4 main groups, in accordance with the National Centre for Complementary and Alternative Medicine [5]. These are divided into mind-body medicine, biologically based therapy, manipulative and body-based practices and the whole medical system. Most of the biological based therapy were based on herbs, either individually or mixed with multiple herbs, honey or animal parts [5]. Manipulative and body-based therapy were the next popular choice of TCM among Malaysians, and under this category included sinusitis treatment, temperature based (cold or hot water application), massage and midwifery [5].

There is clearly growing and widespread use of TCM in Malaysia despite limited data to define the size of TCM industry. In accordance with the Traditional and Complementary Medicine Blueprint 2018–2027, recently published by the Ministry of Health (MOH) Malaysia, 16,050 local TCM practitioners have registered [6]. There were eight TCM practitioner bodies appointed by the MOH prior to enforcement of the Traditional and Complementary Medicine Act. There were approximately 13,000 premises providing TCM services established by the private sectors, and there were more unregistered practitioners estimated nationwide.

The use of TCM is prevalent among patients with chronic or terminal diseases [7]. These include patients with cancer [8–10], heart disease [11, 12] and kidney disease [13–16]. The chronicity of diseases and their limited options for cure by conventional medicine often lead people to turn to TCM [7].

The Kidney Disease Improving Global Outcome Group (KDIGO) has defined chronic kidney disease (CKD) as the presence of abnormalities in kidney structure or function for more than 3 months, with implications for health, and CKD is classified based on cause, glomerular filtration rate (GFR) category, and albuminuria [17]. CKD patients with GFR < 60 mL/min/1.73 m2 are associated with a higher risk of complications, including drug toxicity, metabolic and endocrine complications, especially cardiovascular disease and death. End-stage kidney disease (ESKD) is a state of permanent loss of renal function when measured or calculated GFR is less than 15 ml/min/1.73 m2. In Malaysia, haemodialysis is the most common treatment modality [18]. The total number of haemodialysis and peritoneal dialysis patients in 2016 increased to 35,781 and 3930 patients, giving a prevalence rate of 1159 per million population (pmp) and 127 pmp, respectively [18]. More than double the number of dialysis patients was observed (from 15,087 in 2006), and this number is expected to continue to increase at an alarming rate. ESKD patients on haemodialysis may experience adverse effects physically and mentally. Depression, stress, and other psychological problems are common in this group of patients [19, 20]. Existing comorbidities, such as diabetes mellitus, hypertension, ischaemic heart disease, mineral bone disease and other psychosocial problems, might compromise the quality of life of these patients [20, 21]. Many of these patients struggle to maintain an acceptable quality of life and turn to TCM to address their unmet needs.

The use of TCM among ESKD patients on haemodialysis is a concern, as herbal medicine seems to pose a greater risk to this population than in the general population. This is mainly due to these patients’ poor excretory function, leading to the accumulation of toxic herbal remedies. Volume overload and hyperkalaemia are other important considerations of herbal medicine in ESKD [22]. The loss of excretory functions in patients with ESKD may compound the toxic effects of certain herbal medicines [16], which might not be safe and cause harm to the patients. Several herbs are well known to be associated with worsening kidney disease and might induce harm to consumers with kidney diseases. The European Community introduced a list of unacceptable herbs and made adverse event reporting mandatory in 2004 [23]. The source and composition of these medicines may vary in different parts of the world, depending on cultural or traditional beliefs. However, most of the time, patients were not well informed regarding the proper use of these medicines, resulting in incorrect usage, overdoses, counterfeit herbal medicines and unintentional injuries [1]. Although therapies involving these agents have shown promising potential, with the efficacy of a good number of herbal products clearly established, many of them remain untested, and their use is either poorly monitored or not even monitored at all. The consequence of this is inadequate knowledge of their mode of action, potential adverse reactions, contraindications and interactions with pharmaceuticals [24].

Knowledge of TCM use by ESKD patients is paramount in the tailoring of clinical management, the development of policies, and their implementation and enforcement by regulatory bodies. This study aims to evaluate the use of TCM among chronic haemodialysis patients in Malaysia.

## Methods

This cross-sectional study was conducted in randomly selected private haemodialysis centres throughout Malaysia. The use of TCM among the haemodialysis patients was assessed. This study was conducted over a period of 5 months from 1 April until 31 August 2019. The data collection instrument was a validated questionnaire containing 20 questions on demographics, disease-related characteristics, and various aspects of TCM use, such as types of TCM products used, reasons for use, perceived benefits, and influences and effects of TCM. Face-to-face interviews were conducted with consenting patients during their dialysis sessions. For haemodialysis centres located outside Selangor, Kuala Lumpur, Negeri Sembilan, Melaka and Johor, trained personnel with medical qualifications conducted the interviews.

Inclusion criteria included being older than 18 years old, having been on maintenance haemodialysis for more than 2 years, being able to communicate verbally, being mentally competent (no cognitive or behavioural problems) and being able to give informed consent. There is no universally used definition of chronic haemodialysis in literature review. We had excluded patients less than 2 years on haemodialysis, merely as we thought that 2 years is a good time for patients to be significantly stable with the mode of dialysis and any decision with regards to treatment choice is not influenced easily by their emotional and dynamic decision, compared to those at the early stage of initiation of kidney replacement therapy. Exclusion criteria included refusing to provide consent, having confusion, being unable to complete the interview and having cancer.

### Population

The total number of haemodialysis patients reported in 2016 was 35,781, giving a prevalence rate of 1159 per million population (pmp), based on the 24th report of the Malaysian Dialysis and Transplant Registry in 2016 [18]. A simple random sampling method was used. A list of the private haemodialysis centres in each state in Malaysia was obtained, and the centres were randomly selected using M. S Excel method. From the sampling, the list of selected haemodialysis centres was finalised, and consent was taken accordingly from the management of the respective centres. From the consenting centres, respondents meeting the inclusion and exclusion criteria were selected.

### Sample size calculation

A single proportion formula was used to calculate the prevalence of the study population. The sample size estimation was based on Al-Naggar et al. [8], and PS software was used. A 20% addition to the sample size was included due to the possibility of data entry error and outliers. The total sample required was *n* = 272.

### Questionnaire

The validated questionnaire used in this study has been used in research involving cancer patients in Malaysia [8]. The survey was utilized to obtain information on sociodemographic characteristics and TCM use. Comorbidities and sociodemographic data, including age, sex, ethnicity, and duration of haemodialysis, were obtained through this questionnaire. This validated questionnaire contains 20 questions on demographics, the use of TCM and factors associated with its usage.

### Data collection

Face-to-face interviews were conducted with the patients who agreed to participate in this study. All cases were identified by the code number. The information collected was based on the PROFORMA checklist. These data were entered in a secure database accessible only to the researcher.

### Data analysis

Data were transformed in Microsoft Excel 2013, and statistical analysis was conducted using SPSS version 23 software with descriptive and inferential methods. The descriptive methods included frequency distribution tables and graphs, and the inferential methods included tests of equality of proportions and χ2 tests for associations between the use of TCM and selected sociodemographic and other variables (Fisher’s exact test, the Pearson chi-square test, and the independent t-test were used as applicable). Binary logistic regression was used to identify the predictors of TCM use in the study population based on significant associations identified from sociodemographic variables. All hypotheses were tested at the 5% level of significance. Analysed data are presented as odds ratios (ORs), 95% confidence intervals (95% CIs) and *p*-values. For multiple logistic regression, only variables with p-values < 0.25 or any clinically significant factor were selected for multiple logistic regression analysis. Dependent variables were prevalence of TCM use, types of traditional and complementary medicine, reasons for traditional and complementary medicine use and factors associated with TCM use among haemodialysis patients. Independent variables were sociodemographic characteristics (age, sex, ethnicity, marital and educational status) and the presence of comorbidities.

## Results

A total of *n* = 392 patients were screened. Among these patients, 63 were excluded: 44 patients had a dialysis duration of less than 2 years, and 19 patients did not consent. A total of *n* = 329 haemodialysis patients participated in this study; 18.5% (*n* = 61) of all respondents were from non-governmental organization centres. The mean age of the participants was 54.9 (SD + 12.5) years, with the maximum age of 80 while minimum was 19 years old. Most of the participants were Malay (72%), with a slight preponderance of females at 54.7%. Many patients (*n* = 232, 70.5%) had a household income of less than RM3000 (USD 726) a month, with mean household income of Malaysian was RM7901 (USD1913) [25] and had received secondary-level education (*n* = 227, 69.0%). Most of the participants were married (*n* = 261, 79.3%), and the majority were unemployed (*n* = 193, 58.7%). Demographic data are outlined in Table 1.

**Table 1:** Demography of respondents

Demographic	n (%)	Mean (SD)
**Age**		
< 20	1 (0.3)	
21–30	11 (3.3)	
31–40	55 (16.7)	54.9 (12.5)
41–50	103 (31.3)	
51–60	121 (36.8)	
> 60	149 (45.3)	
**Gender**		
Male	180 (54.7)	
Female	237 (72.0)	
**Ethnicity**		
Malay		
Chinese	67 (20.4)	
Indian	23 (7.0)	
Others	2 (0.6)	
**Household Income**		
< RM 3000 (<USD 720)	232 (70.5)	
RM 3000 – 4999 (USD 720 – USD 1199)	49 (14.9)	
RM 5000 – 9999 (USD 1200 – USD 2399)	38 (11.6)	
RM 10,000 – 15,000 (USD 2400 – USD 3600)	7 (2.1)	
> RM 15,000 (>USD 3600)	3 (0.9)	
**Marital Status**		
Single	25 (7.6)	
Married	261 (79.3)	
Widowed	43 (13.1)	
**Education Status**		
None	6 (1.8)	
Primary School	32 (9.7)	
Secondary School	227 (69.0)	
College/University	60 (18.2)	
Professional	4 (1.2)	
**Employment Status**		
Employed	39 (11.9)	
Self-employed	30 (9.1)	
Unemployed	193 (58.7)	
Pensioner	66 (20.1)	
Student	1 (0.3)	

Almost half of the participants had been on dialysis for 2–3 years (47.7%), with the longest duration of dialysis being 19 years. Participants (32.5%) were mainly from haemodialysis centres located in southern Peninsular Malaysia (Negeri Sembilan, Melaka, Johor), followed by central Peninsular Malaysia (Kuala Lumpur, Selangor) at 22.8%. The other regions (northern Peninsular Malaysia (Perak, Pulau Pinang), eastern Peninsular Malaysia (Pahang, Terengganu, Kelantan) and East Malaysia (Sabah, Sarawak)), represented 17.3, 17.0 and 10.3% of the respondents, respectively. The comorbidities of the participants were mainly hypertension (71.7%), diabetes mellitus (62.9%), dyslipidaemia (13.7%) and heart disease (10.3%). The comorbidities and causes of ESKD of the respondents are outlined in Table 2.

**Table 2:** Comorbidities and causes of ESKD (*n* = 329)

Variables	n (%)
**Comorbidities**^a^	
None	25 (7.6)
Diabetes mellitus	207 (62.9)
Hypertension	236 (71.7)
Gouty arthritis	28 (8.5)
Asthma/lung disease	3 (0.9)
Heart disease	34 (10.3)
Other renal disease (glomerulonephritis/polycystic kidney disease/renal calculi, etc.)	19 (5.8)
Autoimmune disease (systemic lupus erythematosus/rheumatoid arthritis, etc.)	7 (2.3)
Dyslipidaemia	45 (13.7)
Other	7 (2.3)
**ESKD Cause**	
Diabetes mellitus	174 (52.9)
Hypertension	46 (14.0)
Polycystic kidney disease	7 (2.1)
Obstructive uropathy	5 (1.5)
Glomerulonephritis	16 (4.9)
Drug induced, e.g., NSAID	40 (12.2)
Traditional and complementary medicine	5 (1.5)
Unknown	36 (10.9)

^a^Some respondents have multiple comorbidities.

Among the different types of TCM users, the mean age for the group that had never used TCM (56.7 ± 12.3 years) was higher than that for the group that had used TCM before starting dialysis (55.3 ± 12.0 years) and after the initiation of dialysis (51.1 ± 13.1 years) (*p* = 0.015). Primary school educational level had a significant association with TCM use (*p* = 0.009). Other sociodemographic factors did not show any significant correlations with TCM use. These findings are summarized in Table 3.

**Table 3:** Comparison of patient demographics between types of TCM users (*n* = 329)

	Use before dialysis ***n*** = 132 (40.1%)	Use after dialysis ***n*** = 81 (24.6%)	Never use ***n*** = 116 (35.3%)	p- value
**Age (numerical)**	55.3 (12.0)^b^	51.5 (13.1)^b^	56.7 (12.3)^b^	**0.015**^c^
**Age (category)**				
< 20	0 (0.0)	1 (1.2)	0 (0.0)	
21–30	4 (3.0)	4 (4.9)	3 (2.6)	
31–40	13 (9.8)	12 (14.8)	13 (11.2)	–
41–50	24 (18.2)	17 (21.0)	14 (12.1)	
51–60	43 (32.6)	25 (30.9)	35 (30.2)	
> 60	48 (36.4)	22 (27.2)	51 (44.0)	
**Gender**				
Male	72 (54.5)	42 (51.9)	66 (56.9)	0.787^**a**^
Female	60 (45.5)	39 (48.1)	50 (43.1)	
**Ethnicity**				
Malay	95 (72.0)	61 (75.3)	81 (69.8)	
Chinese	27 (20.5)	10 (12.3)	30 (25.9)	0.066^**a**^
Indian	9 (6.8)	10 (12.3)	4 (3.4)	
Others	1 (0.8)	0 (0.0)	1 (0.9)	
**Household Income**				
< RM 3000 (<USD 700)	93 (70.5)	54 (66.7)	85 (73.3)	
RM 3000 – 4999 (USD 700 – USD 1199)	20 (15.2)	12 (14.8)	17 (14.7)	
RM 5000 – 9999 (USD 1200 – USD 2399)	15 (11.4)	12 (14.8)	11 (9.5)	0.981^**a**^
RM 10,000 – 15,000 (USD 2400 – USD 3599)	3 (2.3)	2 (2.5)	2 (1.7)	
> RM 15,000 (>USD 3600)	1 (0.8)	1 (1.2)	1 (0.9)	
**Marital Status**				
Single	8 (6.1)	10 (12.3)	7 (6.0)	
Married	107 (81.1)	62 (76.5)	92 (79.3)	0.480^**a**^
Widowed	17 (12.9)	9 (11.1)	17 (14.7)	
**Education Status**				
None	5 (3.8)	0 (0.0)	1 (0.9)	**0.009**^**a**^
Primary School	18 (13.6)	1 (1.2)	13 (11.2)	

^a^Fisher’s exact test ^b^Mean (standard deviation) ^c^ANOVA

**Fig. 1:**
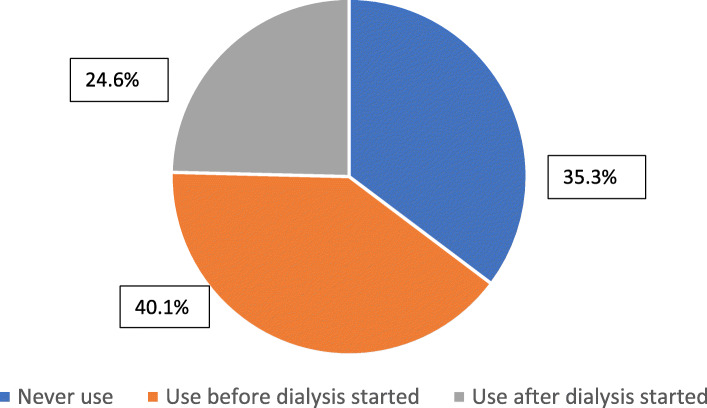
TCM USE

With regard to TCM use, 64.7% (n = 213) of the haemodialysis patients in this study reported TCM use; 40.1% (*n* = 132) of these patients used TCM before the initiation of dialysis, while 24.6% (*n* = 81) used TCM only after the commencement of renal replacement therapy (Fig. 1).

**Fig. 2:**
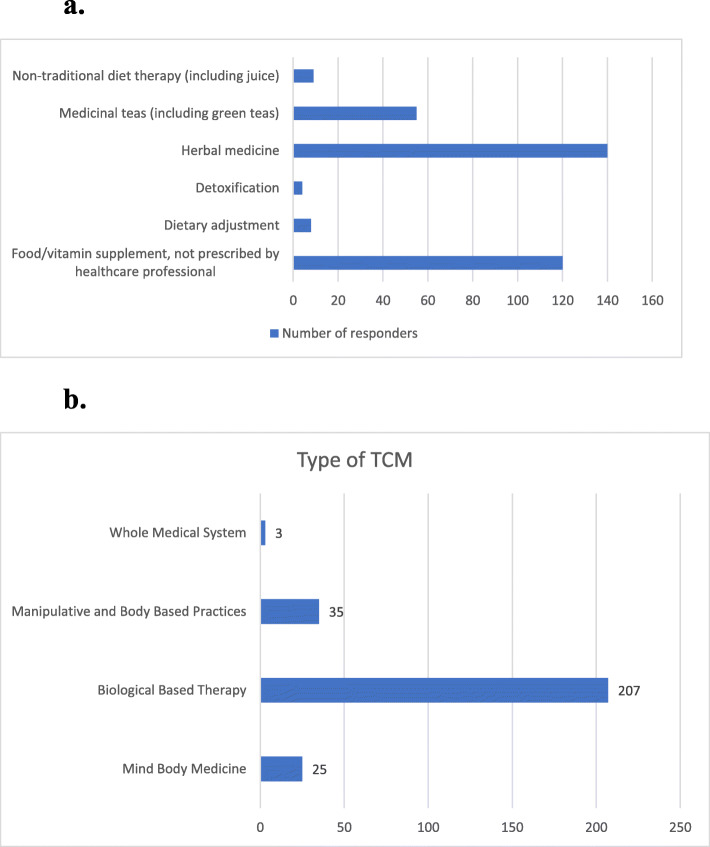
**a** Number of biologically based therapy users based on type (*n* = 207). * Some respondents may have multiple responses**. b** Number of TCM users based on the types (*n* = 213).* Some respondents may have multiple responses

Among the four categories of TCM, biologically based therapy was found to be the most popular and was used by 97.2% (*n* = 207) of the sample. Herbal medicine (67.6%) and supplements that were not prescribed by healthcare professionals (58.0%) were also popular choices. These were outlined in Fig. 2a and b.

**Fig. 3:**
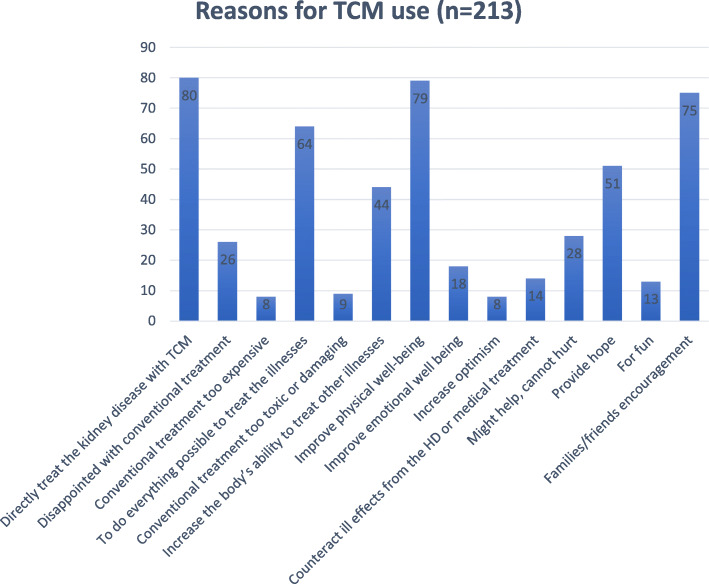
* Some respondents may have several reasons

Most respondents (55.4%) used TCM occasionally, 20.7% used TCM on a weekly basis, 20.2% used TCM daily, and a minority (3.8%) used TCM only once. Friends and other patients (58.7%) were the main sources of information about TCM, followed by family members (42.3%) and the media (39.4%). Reasons for TCM use were to directly treat kidney disease (37.6%) and improve physical well-being (37.1%); additionally, 35.2% of the sample used TCM at the encouragement of family members and friends (Fig. 3).

Many respondents did feel better with the usage of TCM (*n* = 89, 41.8%); and majority reported had no side effects after consumption (*N* = 131, 61.5%). The total amount of money spends were variable. The data with regards to perception, side effects and money spend on the usage of TCM are summarized in Table 4.

**Table 4:** Patient’s Perceived Benefit, Side Effects from TCM and Total Money Spent for TCM (*n* = 213)

Variables	n (%)
**Patient’s Perception of Benefits**	
No effect at all	75 (35.2)
I feel better	89 (41.8)
I feel worse	15 (7.0)
I am not sure	34 (16.0)
**Patient’s Perception of Side Effects**	
Yes^a^	20 (9.4)
No	131 (61.5)
Uncertain	62 (29.1)
**Total Money Spent**	
Less than RM 100 (USD 24)	58 (27.2)
RM 100–299 (USD 24–72)	44 (20.7)
RM 300–499 (USD 72–120)	37 (17.4)
RM 500–999 (USD 120–240)	32 (15.0)
RM 1000–4999 (USD 240–1200)	37 (17.4)
More than RM 5000 (USD 1200)	5 (2.3)

^a^ side effects experienced by patients include vomiting, nausea, itchiness, diarrhea, lethargic and body swelling

The majority (*n* = 154, 72.3%) of participants did not disclose their TCM usage to their managing doctors (Table 5). The reasons were because the doctors did not ask (*n* = 63, 41.2%), patients assumed that their doctors would disapprove (*n* = 51, 33.3%), and patients thought it was not necessary for their doctors to know (*n* = 39, 25.5%). These are summarized in Table 5.

**Table 5:** Disclosure to Doctor (*n* = 213)

Variables		n (%)
**Told doctor about TCM usage**	Yes	59 (27.7)
	No	154 (72.3)
**Why did patient disclose this information?**	Doctor asked	26 (44.1)
	Doctor should know	12 (20.3)
	To ask for the doctor’s opinion	21 (35.6)
**Doctor’s reaction**	In favour	5 (8.5)
	Opposed	40 (67.8)
	No Opinion	14 (23.7)
**Why did the patient not disclose this information?**	Doctor did not ask	63 (41.2)
	Not necessary	39 (25.5)
	Doctor would disapprove	51 (33.3)

**Table 6:** Employment status between user and non-user of TCM

Demographic		n (%)	User n (%)	p-value
**Employment Status**	Unemployed	79 (68.1)	114 (53.5)	**0.010**^**d**^
	Other	37 (31.9)	99 (46.5)

**Table 7:** Logistic Regression – Outcome – TCM user vs nonuser

Variables		Crude B	Crude OR (95% CI)	p-value
**Age (numerical)**		- 0.02	0.98 (0.96, 1.00)	0.056
**Age (categorical)**	< 20	−20.22	–	–
	21–30	−20.55	–	–
	31–40	−20.14	–	–
	41–50	−20.54	–	–
	51–60	−20.89	–	–
	> 60	−20.22	–	–
**Gender**	Male	0	1	
	Female	- 0.14	0.87 (0.55, 1.38)	0.557
**Ethnicity**	Malay	0	1	
	Chinese	- 0.45	0.64 (0.37, 1.11)	0.113
	Indian	0.90	2.47 (0.81, 7.49)	0.111
	Others	- 0.66	0.52 (0.03, 8.41)	0.645
**Household Income**	< RM 3000 (<USD 700)	0	1	
	RM 3000 – 4999 (USD 700 – USD 1199)	0.085	1.09 (0.57, 2.08)	0.797
	RM 5000 – 9999 (USD 1200 – USD 2399)	0.350	1.42 (0.67, 3.01)	0.360
	RM 10,000 – 15,000 (USD 2400 – USD 3599)	0.369	1.45 (0.27, 7.61)	0.664
	> RM 15,000 (>USD 3600)	0.145	1.16 (0.10, 12.94)	0.906
**Marital Status**	Single	0	1	
	Married	- 0.336	0.71 (0.29, 1.77)	0.468
	Widowed	- 0.520	0.60 (0.21, 1.73)	0.339
**Education Status**	None	0	1	
	Primary School	− 1.230	0.29 (0.03, 2.80)	0.286
	Secondary School	−1.020	0.36 (0.04, 3.14)	0.355
	College / University	−.990	0.37 (0.04, 3.39)	0.380
	Professional	19.593	–	–
**Employment Status**	Employed	0	1	
	Self-employed	- 0.053	0.99 (0.32, 2.80)	0.923
	Unemployed	- 0.698	0.50 (0.23, 1.08)	0.077
	Pensioner	- 0.159	0.85 (0.35, 2.09)	0.728
	Student	20.138	–	–
**Variables**		**Crude B**	**Crude OR (95% CI)**	**p-value**
**Comorbid**	No	0	1	
	Yes	- 0.59	0.56 (0.22, 1.44)	0.226
Comorbid – Diabetes mellitus	No	0	1	
	Yes	- 0.12	0.89 (0.56, 1.43)	0.630
Comorbid – Hypertension	No	0	1	
	Yes	- 0.25	0.78 (0.47, 1.30)	0.332
Comorbid – Gouty arthritis	No	0	1	
	Yes	- 0.35	0.70 (0.32, 1.54)	0.381
Comorbid – Asthma/ lung disease	No	0	1	
	Yes	0.09	1.09 (0.10, 12.15)	0.944
Comorbid – Heart disease	No	0	1	
	Yes	- 0.28	0.76 (0.37, 1.56)	0.447
Comorbid – Other renal disease	No	0	1	
	Yes	- 0.07	0.93 (0.36, 2.43)	0.882
Comorbid – Autoimmune disease	No	0	1	
	Yes	0.315	1.37 (0.26, 7.18)	0.709
Comorbid – Dyslipidaemia	No	0	1	
	Yes	0.463	1.59 (0.79, 3.21)	0.197
**Dialysis Duration (categorical)**	2–3	0	1	
	4–5	0.34	1.40 (0.81, 2.42)	0.231
	6–7	< 0.01	1.00 (0.49, 2.06)	0.993
	8–9	- 0.39	0.68 (0.25, 1.85)	0.448
	> 10	0.47	1.61 (0.60, 4.33)	0.350
**Dialysis Duration (numerical)**		0.019	1.02 (0.94, 1.11)	0.654
Location (Region)*	North Peninsular	0	1	
	East Peninsular	- 0.108	0.90 (0.40, 2.00)	0.790
	South Peninsular	- 0.725	0.49 (2.5, 0.96)	**0.038**
	Central Peninsular	0.531	1.70 (0.76, 3.79)	0.194
	East Malaysia	- 0.738	0.48 (0.20, 1.15(	0.101

Note:

No variable that have significant association with TCM user (or non-user)

Some cells have small size (count < 5), making crude B coefficient to be very large (either < − 5 or > 5), and cannot be interpreted

Post hoc analysis with Bonferroni adjustment showed that there was a significant difference in mean age between the group that had never used TCM and the group that had used TCM after the initiation of dialysis. The mean age was found to be significantly higher (*p* = 0.015), with a difference of 5.1 years (SE = 1.8). In comparing users and non-users of TCM, there was no significant association with the users of TCM. Employment status did not show any significant correlation when it was divided into subgroups of employed, self-employed, unemployed, pensioner and student. However, there was statistically significant association between employment status of TCM user when it was subgroup into unemployed vs others (employed, self-employed, pensioner and student) (*p* = 0.01). The odd ratio of non TCM user being unemployed compared to the other groups was 1.85 (95% CI: 1.15, 2.98). Tables 6 and 7 summarize these findings.

## Discussion

To the authors’ knowledge, this is the first study examining the TCM usage and practices of chronic haemodialysis patients in Malaysia. This was a multi-centre cross-sectional survey that examined n = 329 participants, a representative of the dialysis population nationwide. The results demonstrated that 64.7% of all participants reported common use of TCM; the majority did so without informing their managing healthcare professionals and used TCM before the initiation of dialysis. Commonly used types of TCM are herbal treatment and non-prescribed supplementation.

The overall age group that used TCM corresponded with the findings of published studies from India and the United States of America (USA). Similarly, it was shown that the usage of TCM was high in the group of patients 50–64 years of age [13, 26]. Our report revealed that patients who used TCM after the initiation of dialysis were younger (51.1 ± 13.1 years) than the patients who used TCM before the initiation of dialysis (55.3 ± 12.0 years). However, an association between age and TCM use was not found in other published studies [13, 15]. For this study, most of the respondents were Malay, as they are the ethnic majority in Malaysia. No association was found between race and TCM use; this agrees with the findings of previously published research [13, 15].

Our study revealed that patients with primary education and below were unlikely to use TCM after the initiation of dialysis. A possible explanation could be that those groups were more adherent to the treatment prescribed to them, rather than resorting to alternative therapy. People with higher educational levels harbour more cynicism towards conventional medicine, and they are more aware of TCM, as noted in a previously published finding [8]. Unemployment was more likely to be a characteristic of a non-TCM user than were other characteristics (employed, self-employed, pensioner and student). Financial stability and availability may enable patients to try alternative therapies. However, the results in the literature are conflicting, as prior published studies have found no association between educational level, employment and TCM use [13, 15, 26]. A larger sample size is needed to examine this finding in more detail.

The prevalence of TCM usage among chronic haemodialysis patients was 64.7%, with the majority consumed TCM before the commencement of haemodialysis. Our inclusion criteria included chronic haemodialysis patients undergoing treatment for more than 2 years in duration. We did not find an association between dialysis vintage and usage of TCM; the findings are similar to other studies [13, 15, 27]. The use of TCM pre-dialysis may pose a risk to these groups of patients. Patients with chronic kidney disease (CKD) may believe that TCM may slow the progression of their renal disease since it is nature-based and assume that they can use it without obtaining further medical advice. The prevalence of TCM use among chronic haemodialysis patients in Malaysia was similar to the prevalence shown in data from Palestine (64.4%), with herbal medicine being the most popular choice of TCM [27]. Other countries reported a lower prevalence, e.g., India (26%), with Ayurveda being reported as the most commonly used TCM (30.4%) [26]. The prevalence of TCM use among chronic kidney disease patients in Turkey was 25.2% and primarily consisted of mind-body therapies (46.1%) [28]. In Trinidad, the prevalence of TCM use among chronic haemodialysis patients was only 18.8%, with mostly used medicinal herbs used [15]. The prevalence varies as TCM use differs according to cultural beliefs and practices. Among the general population in Malaysia, the prevalence of TCM use was reported to be 69.4% [5]. Our results supported the findings of this study and demonstrated that Malaysia has one of the highest prevalence estimates of TCM use worldwide. Future research needs to examine the reasons underlying this phenomenon.

The majority of the respondents did not disclose their usage of TCM to the doctors treating them because the doctors did not ask the patients about it. A third refused to disclose, as they knew that doctor would disapprove, while the rest thought that it was not necessary for doctors to know. In the USA, approximately one-third of patients use herbal products without informing their doctors [29, 30]. In Australia, the majority of patients use TCM, including herbal medicines, without the knowledge of their doctors, and the products are often used with conventional medicine [31, 32]. With reported side effects and potential harm, this unsafe practice needs to be addressed. Healthcare professionals will need to heighten activities on patient education and awareness. More research is needed to examine the reasons for these decisions and explore areas that can improve the situation.

Biologically based therapy was found to be the most popular choice among chronic haemodialysis patients, which included herbal medicines, supplements and medicinal teas. These findings reflect those of previously published data on the general population in Malaysia demonstrating herbal therapy as the most used form of TCM [5]. However, for cases in the post-stroke cohort, different forms of TCM (acupuncture and massage) were found to be the two most popular methods [33]. The use of herbal medicines is very alarming, as ESKD patients are more susceptible to their detrimental effects than are those in the general population [16]. This greater susceptibility among ESKD patients is mainly due to the loss of renal function, which results in the accumulation of toxic materials in the system that cause harm to the patients. Herbal products may pose problems, as their ingredients may affect and interact with prescribed medications. They may contain undeclared ingredients, lack evaluation according to appropriate medical standards, undergo inadequate processing and be subject to non-disclosure of usage [10]. The unpredictable pharmacokinetics, drug interactions, nephrotoxicity, haemodynamic alterations, and adverse effects on blood pressure, blood glucose, or electrolyte abnormalities make ESKD patients more vulnerable [34].

Biologically based therapies, namely, herbal products and supplements, are very popular on the market, are easily accessible and can be bought online. People may believe that these products are safer than prescribed drugs are because they perceive that nature-based products are safer for consumption. Most of the participants claimed that they felt better with the use of TCM. TCM was taken concomitantly with conventional treatment. Hence, it may be difficult to differentiate between various treatment effects. A more comprehensive RCT will need to be performed to address this question. Most participants reported using TCM to directly treat their kidney problems and improve physical well-being; other reported using TCM because they were encouraged by family or friends to do so. One-fifth of the patients use TCM on a daily basis. The findings of this study confirmed that the majority of ESKD patients have high trust in TCM. Despite the establishment of the Traditional and Complementary Medicine Council in many countries to regulate traditional and complementary medicine services [1, 35], the use of TCM has yet to be properly regulated. The findings of this study demonstrated the extent of TCM use in vulnerable populations in Malaysia. Since safety has been recognized as a major issue with the use of herbal remedies, it becomes imperative that relevant regulatory authorities establish appropriate measures to protect public health and improve enforcement efforts.

Several limitations of this study were recognized. This is only a cross-sectional study; hence, it does not take into account disease trajectory. This survey is subject to recall bias. The haemodialysis centres that were chosen may not be representative, as they were randomly selected, and many were in major cities.

## Conclusions

TCM use is a common practice among chronic haemodialysis patients in Malaysia, especially herbal medicine and supplements. Age, educational level and employment status were found to have a significant association with usage of TCM. We observed that patients that never use TCM has higher mean of age, compared to the group that use TCM after being initiated on dialysis. Patients who received lower than primary education tend not to be TCM users after initiation of dialysis compared to those with higher education, and unemployment was found to be more likely associated with non TCM use. There is a lack of disclosure to healthcare professionals regarding TCM use, and there is a need for healthcare professionals to be more vigilant in obtaining information.

## Supplementary Information

**Additional file 1.**

## Data Availability

The datasets used and/or analysed during the current study available from the corresponding author on reasonable request..

## Abbreviations

CKD: Chronic Kidney Disease
ESKD: End Stage Kidney Disease
TCM: Traditional and complementary medicine
