# Supplementary material for: Traditional and complementary medicine use among chronic haemodialysis patients: a nationwide cross-sectional study
Source: BMC Complement Med Ther. 2021 Mar 16;21:94. doi: 10.1186/s12906-021-03268-4 (PMC7967981; doi:10.1186/s12906-021-03268-4)
Supplement: Supplementary file 1 — Additional file 1. [file 12906_2021_3268_MOESM1_ESM.pdf]

APPENDICES

Appendix 1A (Questionnaire- English version)

Traditional and Complementary Medicine (TCM) Use Among Chronic Haemodialysis Patients in Malaysia

This questionnaire is designed to find out to what degree haemodialysis (HD) patients in Malaysia use alternative and complementary medicine. It is only a research work. The findings of this research will help us to understand our HD patients better and therefore help us to improve the care that we render to them. We implore you to give us as honest an answer to each question as possible. You may choose not to respond to any of the questions you are not comfortable with. We guarantee you confidentiality.

Haemodialysis centre and its location (State)

____

1. How long have you been on dialysis? (Please state answer in years)

____

2. Date of birth: __/__/____

3. Sex ◻ Male ◻ Female

4. Household income:

Less than RM 3000	
RM 3000 – RM 4999	
RM 5000 – RM 9999	
RM 10000 – RM 15000	
More than RM 15000	

5. Marital status:

Single	
Married	
Divorced/Spouse already passed away	

6. Highest level of education:

None	
Primary school	
Secondary school	
College/University	
Professional degree	
Other (please specify)	

7. Employment status

Employed	
Self - employed	
Not employed	
Pensioner	
Studying	

8. Race

Malay	
Chinese	
Indian	
Others (please specify)	

9. Comorbidity:

Diabetes mellitus	
Hypertension	
Gouty arthritis	
Asthma/ lung disease	
Heart disease	
Other renal disease [Glomerulonephritis/Polycystic kidney disease/Renal calculi etc]	
Autoimmune disease [Systemic lupus erythematosus/ Rheumatoid arthritis etc)	
Dyslipidaemia	
None	
Other (please specify)	

10. Cause of ESRD:

Diabetes mellitus	
Hypertension	
Polycystic kidney disease/Obstructive uropathy	
Glomerulonephritis	
Drug induced e.g. NSAID	
Traditional and complementary medicine	
Unknown	
Other	

11. Traditional and complementary medicine (TCM) use

TCM use since before started on dialysis	
No TCM use before dialysis, only after started on dialysis	

Never use TCM	

(Proceed with question 12 if you have been using or had use TCM)

12. Please check all that you are currently using or have/had been using in the past (please check all that apply)

Food/vitamin supplement that was not prescribed by healthcare professional	
Dietary adjustment	
Detoxification	
Acupuncture	
Massage	
Aromatherapy	
Electromagnetic therapy	
Spiritual therapies	
Herbal medicine	
Meditation	
Tai chi	
Yoga	
Medicinal teas (including green teas)	
Non-traditional diet therapy (including juice)	
Hypnosis	
Mental imagery	
Psychic therapy	
Reflexology	
Bloodletting Cupping	
Other (please specify)	

13. Reasons for using traditional and complementary medicine (TCM)

Directly treat the kidney disease with TCM	
Disappointed with conventional treatment	
Conventional treatment too expensive	
To do everything possible to treat the illnesses	
Conventional treatment too toxic or damaging	
Increase the body’s ability to treat other illnesses	
Improve physical well-being	
Improve emotional well being	
Increase optimism	
Counteract ill effects from the HD or medical treatment	
Might help, cannot hurt	
Provide hope	
For fun	

Families/friends encouragement	
Other (please specify)	

14. How frequently have you been using traditional and complementary medicine (TCM)?

Daily	
Weekly	
Occasionally	
Only once	

15. Source of information regarding TCM (check all that apply)

Local newspaper/magazine	
Foreign newspaper/magazine	
Television/ radio	
Internet/ Social media	
Healthcare professionals	
Other patients	
Friends	
Family members	
Health Talk	
Religious contacts	
Practitioners of TCM	
Other (please specify)	

16. When using these supplements or alternative therapies, have they benefited you?

No effect at all	
I feel better	
I feel worse	
I am not sure	

17. When using these supplements or alternative therapies, have you experienced unpleasant side effects?

Yes, please specify	
No	
Uncertain	

18. About how much money have you spent on TCM?

Less than RM 100	
RM 100 – RM 299	
RM 300 – RM 499	
RM 500 – RM 999	
RM 1000 – RM 4999	

More than RM 5000	

19. Have you told your doctor about these supplements or alternative therapies?

Yes, because

Doctor asked	
Doctor should know	
Wanted to know the doctor's opinion	
Other (please specify)	

No, because

Doctor did not ask	
It was not necessary for the doctor to know	
Doctor would disapprove	
Other (please specify)	

20. If you told your doctor, what was his/her reaction? (check all that apply)

Doctor in favour	
Doctor opposed	
Did not offer opinion	
Other (please specify)	

Thank you
